# Symptoms of anxiety but not depression before start of taxane-based chemotherapy are associated with peripheral neuropathy: a multicenter study in women with breast cancer

**DOI:** 10.1007/s00520-022-07093-4

**Published:** 2022-05-11

**Authors:** Rita Verhoeff-Jahja, Moniek M. ter Kuile, Nir I. Weijl, Rianne Oosterkamp, Marissa Cloos, Johanneke E. A. Portielje, Judith R. Kroep, Chris Hinnen

**Affiliations:** 1 Oncology Centre, Psycho-Oncology, Leiden University Medical Center, Albinusdreef 2, Leiden, The Netherlands; 2 Gynaecology Department, Leiden University Medical Center, Leiden, The Netherlands; 3 Department of Medical Oncology, Haaglanden Medical Center, The Haque, The Netherlands; 4 Department of Oncology, Groene Hart Hospital, Gouda, The Netherlands; 5 Department of Oncology, Leiden University Medical Center, Leiden, The Netherlands

**Keywords:** Chemotherapy-induced peripheral neuropathy, Cancer, Anxiety, Depression

## Abstract

**Background:**

Chemotherapy-induced peripheral neuropathy (CIPN) is a common side effect of chemotherapy, especially after taxane-based therapy. This study aimed to examine the relationship between symptoms of anxiety and depression before the start of taxane-based chemotherapy and the development of CIPN in women with breast cancer.

**Methods:**

In this prospective study, women with breast cancer receiving taxane-based (neo)adjuvant chemotherapy were recruited from four hospitals in the Netherlands. Patients completed questionnaires assessing anxiety and depressive symptoms before treatment and CIPN before treatment (T0), 6 weeks after start of treatment (T1), after the last cycle of chemotherapy (T2), and 6 months after the end of treatment (T3). Mixed model analyses were used to investigate whether medium/high levels of anxiety or depression at baseline are associated with the level of CIPN during and after treatment.

**Results:**

Among the 61 participating women, 14 (23%) reported medium/high levels of anxiety and 29 (47.5%) reported medium/high levels of depressive symptoms at baseline. The group of women with medium/high baseline levels of anxiety showed a significantly higher increase in CIPN during and after chemotherapy than women with low baseline levels of anxiety (*p* < .001). No relationship between depressive symptoms at baseline and the development of CIPN was found.

**Conclusion:**

This study showed that baseline medium to high levels of anxiety but not depressive symptoms impacted the development of CIPN during and in the 6 months after treatment.

## Background

Chemotherapy has contributed significantly to the increased survival rates in women with breast cancer. However, chemotherapy, especially taxane-based, may have long lasting adverse side effects such as peripheral neuropathy (CIPN). CIPN is characterized by symptoms such as numbness, tingling, pins and needles sensation, hyperalgesia or allodynia, and burning pain in the hands or feet in a stocking-glove distribution [1–3]. CIPN can negatively affect daily functioning and quality of life [4] and may impact optimal treatment [5, 6]. To date, there are no effective interventions or agents available to prevent or treat CIPN [7]. Consequently, dose reduction or chemotherapy cessation may be necessary to prevent severe CIPN [8–10].

The reported prevalence of CIPN in cancer patients treated with taxane-based chemotherapy differs from 11 to 87% [11]. Also, the variability in CIPN severity and duration is considerable, ranging from mild to severe and from temporal to chronic. CIPN generally develops during chemotherapy and may persist after treatment. The variations in intensity, development, and duration are not well understood and are likely to be multifactorial by nature [12]. Characteristics such as age, BMI, diabetes, preexisting peripheral neuropathy, and the influence of cumulative dose are found to increase the risk of developing CIPN [4, 13, 14]. Besides these sociodemographic and clinical characteristics, psychological factors may play a role as well. Previous research pointed to an association between anxiety and CIPN in cancer survivors after chemotherapy [5, 15]. Lee and colleagues (2018) found in their prospective observational study among women with breast cancer that pre-treatment anxiety but not depressive symptoms was associated with elevated neuropathic symptoms directly after ending treatment, and this effect persisted after 8 months. A limitation of this study was that no data were available of CIPN during treatment and therefore it remains unclear when differences in CIPN start to occur. Moreover, in this study, no validated method to assess CIPN was used.

The primary aim of the present study was to investigate the relationship between symptoms of anxiety and depression and the level and development of CIPN during and after taxane-based (neo)adjuvant chemotherapy in women with breast cancer. Nonindependence of the scores of individual participants over time were taken into account using mixed model analyses. We hypothesized that the increase of CIPN during and after chemotherapy is enhanced in patients who report medium to high levels of anxiety before the start of taxane-based chemotherapy. No relationship between symptoms of depression and CIPN was expected.

## Methods

### Study design and patient population

Participants were enrolled in this prospective observational study in four hospitals in the Netherlands (Leiden University Medical Centre, Groene Hart Hospital, Haga Hospital, and Alrijne Hospital) between January 2020 and September 2021. Participants were women with breast cancer, aged 18 years or older, treated with taxane-based (neo)adjuvant chemotherapy. Exclusion criteria were obvious cognitive impairments and not speaking Dutch well enough to participate in the assessments. Eligible participants completed baseline questionnaires before the first cycle of chemotherapy, recording socio-demographic as well as symptoms of anxiety and depression. CIPN was measured before start of chemotherapy (T0), after 6 weeks (T1), after the last cycle of chemotherapy (after 15 weeks, T2), and 6 months after ending chemotherapy (T3). Clinical data (i.e., cancer grade, chemotherapy regimen, discontinuation of treatment) were extracted from patients’ medical records. The NUMBNESS study was approved by the certified Medical Ethical Committee Leiden-Den Haag-Delft (METC-LDD, registration number: N19.111). Written or digital informed consents were obtained from all patients.

### Sociodemographic and clinical characteristics

Before the start of chemotherapy, information was provided on sociodemographic characteristics such as age, marital status, household composition, education level, weight and height, smoking, and alcohol use by self-report questionnaires. Also information was obtained on the presence of diabetes or rheumatism, use of antidepressants, and having received chemotherapy in the past. Tumor stage (TNM staging system), type of taxane regiment, and information about discontinuing the chemotherapy were obtained from patients’ medical files.

### Primary outcome: neuropathic symptoms

The European Organization for Research and Treatment of Cancer Quality of life Questionnaire Chemotherapy-Induced Peripheral Neuropathy 20 (EORTC QLQ-CIPN20) was used to assess CIPN. Patients were asked at four time points to indicate how often they had experienced peripheral neuropathic symptoms in the past week. Nineteen items were answered on a four-point Likert scale ranging from (1) not at all to (4) very much. Item 20 is a male-related question and was not included in the questionnaire. Item 19 can only be answered by patients who can drive a car. Total scores of the CIPN20 are used and range from 18 to 76, with higher scores indicating more neuropathy. Cronbach’s alpha of the EORTC QLQ-CIPN20 at the four time points was 0.88 (T0), 0.75 (T1), 0.87 (T2), and 0.95 (T3).

### Secondary dependent variable: symptoms of anxiety and depression

Anxiety was measured by the Generalized Anxiety Disorder (GAD-7) [16]. The GAD-7 contains 7 items assessing core anxiety symptoms. Patients rate their frequency of symptoms on a 4-point Likert scale ranging from 0 (not at all) to 3 (almost every day) over the previous 2 weeks. GAD-7 scores can range from 0 to 21, with higher scores indicating higher anxiety symptomatology. The cut-off point for the presence of moderately/high anxiety symptoms is 5 [17]. Cronbach’s alpha of the GAD-7 was 0.86.

Depression was measured by the Patient Health Questionnaire (PHQ-9). The PHQ-9 contains 9 items to score the symptoms of depression on a 4-point Likert scale ranging from 0 (not at all) to 3 (almost every day) over the previous 2 weeks. The total PHQ-9 scores can range from 0 to 27, with higher scores indicating more depression symptoms. The cut-off point for the presence of moderately severe depressive symptoms is 5 [18]. Cronbach’s alpha of the PHQ-9 was 0.76.

### Statistical analysis

Baseline characteristics were determined and associated with CIPN using Pearson’s product correlation coefficients, independent sample *t*-test, or one-way ANOVA analyses. Variables significantly (*p* < .05) associated with CIPN at baseline were used as covariates in the main analyses. Moreover, when baseline anxiety or depression is associated with baseline CIPN, baseline CIPN will be included as covariate in the main analyses. By controlling for the baseline CIPN levels, it is possible to investigate the effect of baseline anxiety or depression on the developing CIPN during and after treatment, independent of the CIPN baseline levels.

Mixed models with maximum likelihood estimation and an unstructured covariance matrix with a 2-level structure (time-level and patient-level) were used. Mixed model analyses allow the number of observations per assessment to differ and therefore missing data were not imputed. A sequence of models was fitted to investigate the development of CIPN during and in the first 6 months after chemotherapy and whether this is influenced by the level of anxious or depressive symptoms at baseline. First, a model with no explanatory variables, only the intercept (i.e., an unconditional model), was calculated to determine the amount of variance at the person and time level. Second, a model with only time as explanatory variable (unconditional growth model) was calculated to determine whether CIPN changed from pre-treatment till 6 months after ending chemotherapy. Intercept and time are entered both as fixed and random effects as each subject may have its own unique intercept and slope. Third, the unconditional growth model was extended into a conditional growth model by including (i) possible covariates (i.e., age, BMI, receiving chemotherapy in past) and baseline CIPN, (ii) baseline level of anxiety or depression, and (iii) the interaction term of time by level of anxiety or depression as fixed effect. By including the interaction term, we were able to investigate whether CIPN develops differently over time depending on baseline levels of anxiety and depression. Regression estimates and 95% confidence interval of fixed effects and the Akaike information criteria (AIC) are presented. A model-based graph was created as an aid to determine how the relationship between anxiety and depressive symptoms and CIPN could be understood.

## Results

### Patients’ sociodemographic and clinical characteristics

In total, 63 eligible patients were included. Two patients were excluded from further analyses because they did not finish the chemotherapy due to an allergic reaction. Of the remaining 61 women, 59 completed assessment 6 weeks after starting chemotherapy (T1), 56 after the last cycle of chemotherapy (T2), and 36 completed assessment 6 months after ending chemotherapy (T3). Table 1 shows baseline clinical and demographic characteristics. At inclusion, mean age was 51.7 years (SD 10.4; range 25.0–75.0) and mean BMI was 26.4 (SD 4.9; range 19.9–42.1).

**Table 1:** Sociodemographic and clinical characteristics of participants (*n*=62)

*Characteristics*	*Mean (SD) or number (%)*
Age	51.7 (10.4)
BMI (kg/M^2^)	26.4 (4.9)
Tumor stage (TNM staging system)	
I	14 (23.0%)
II	30 (49.2%)
III	13 (21.3%)
Regiment	
4× dd AC, 12× weekly paclitaxel	44 (72.1%)
Only 12× weekly paclitaxel	4 (6.6%)
4× Docetaxel (q3 weeks)	1 (1.6%)
Carboplatin d1, paclitaxel d1,8, trastuzumab d1, pertuzumab d1, q3 weeks	7 (11.5%)
12x weekly paclitaxel, trastuzumab	3 (4.9%)
Other regiment	2 (3.3%)
Chemotherapy in past (yes)	16 (26.2)
Smoking (yes)	3 (4.9%)
Diabetes (yes)	2 (3.3%)
Rheumatism (yes)	1 (1.6%)
GAD-7 ≥ 5 at baseline	14 (23%)
PHQ-9 ≥ 5 at baseline	31 (48.4%)

*BMI*, body mass index; *TNM*, the T refers to the size and extent of the main tumor, the N refers to the number of nearby lymph nodes that have cancer, and the M refers to whether the cancer has metastasized; *dd*, dose dense; *AC,* adriamycin and cyclophosphamide; *GAD-7,* Generalized Anxiety Disorder Assessment; *PHQ-9,* Patient Health Questionnaire

Nine percent of the patients were diagnosed with stage II disease and most patients received treatment with weekly paclitaxel (84%, *n*=51). Sixteen patients (26.2%) had received treatment with chemotherapy in the past, all other than taxane based. Baseline characteristics such age, BMI, smoking, diabetes, rheumatism, and chemotherapy in the past were not found to be associated with CIPN levels before start chemotherapy. At baseline, 23% (*n*=14) of the women reported moderately/high anxiety symptoms and 48.4% (*n*=31) reported moderately/high depressive symptoms. Baseline anxiety was found to be associated with baseline CIPN (*p* = .001). That is, patients with a low anxiety score had a baseline CIPN score of 21.1 (SD = 3.17) and high anxiety score had baseline CIPN score of 25.93 (SD = 8.28). No association was found between baseline depression and baseline CIPN (*p* =.07).

### Association between CIPN, time, and baseline level of anxiety and depression

Intraclass correlation coefficients of the unconditional model showed that 43% of the variance was at the person level and the remaining 57% was at the time level. These results indicate that scores on CIPN differed enough between patients and over time to justify a two-level model (AIC = 1398.59). Next, we computed an unconditional growth model by entering time into the model. This model fitted the data better than the unconditional model (AIC = 1351.60) and showed that CIPN increases over time from a mean score of 22.25 at T0 (95% CI 20.58–23.92) to 28.46 at T3 (95% CI 26.48–30.45). Moreover, the conditional growth model with time, level of anxiety at baseline, and the interaction term time by anxiety was computed. This model fitted the data better than the unconditional growth model (AIC = 1300.01). No baseline characteristics such as BMI, age, smoking, and diabetes were included as these were found not to be significantly associated with baseline CIPN. This model (see Table 2) shows that time, baseline CIPN, and baseline level of anxiety had an impact on level of CIPN.

**Table 2:** Fixed effects of time, baseline CIPN, and level of anxiety on CIPN over time

	Estimate	95% CI	*p*
Intercept^a^	20.70	15.14–26.28	< .001
Baseline CIPN^b^	.70	.53–.87	<.001
T0 (pre-treatment)	−12.91	−16.67 to −9.14	<.001
T1 (6 weeks after start)	−12.41	−16.17 to −8.64	<.001
T2 (post-treatment)	−6.91	−10.67 to −3.14	<.001
Low anxiety	−10.31	−14.11 to −6.52	< .001
T1*Low anxiety	8.87	4.61 to 13.13	<.001
T2*Low anxiety	10.14	5.88 to 14.41	<.001
T3*Low anxiety	8.17	3.89 to 12.45	<.001

^a^CIPN at T3 for patients with moderate to severe baseline anxiety

^b^Baseline CIPN is set at 22.29

**Fig. 1:**
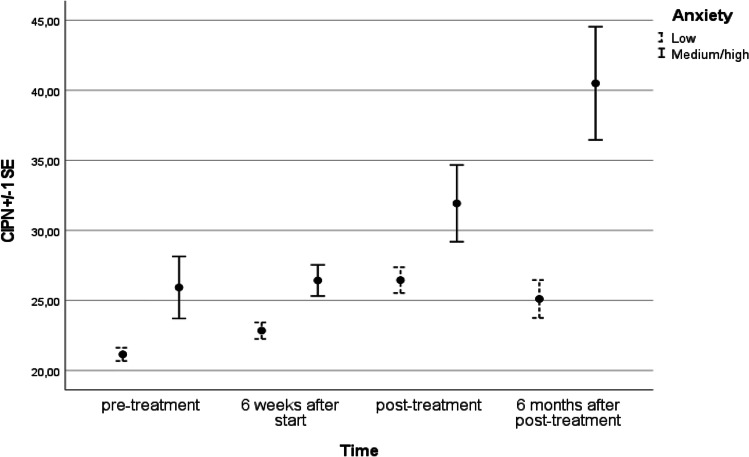
CIPN levels at 4 time points for group with low and medium/high anxiety at baseline

The mean difference in CIPN between patients scoring low and medium/high on baseline anxiety was 3.52 (*p* =.002). Moreover, also the development of CIPN over time was found to differ between patients scoring low and medium/high on baseline anxiety (see also Figure 1).

**Fig. 2:**
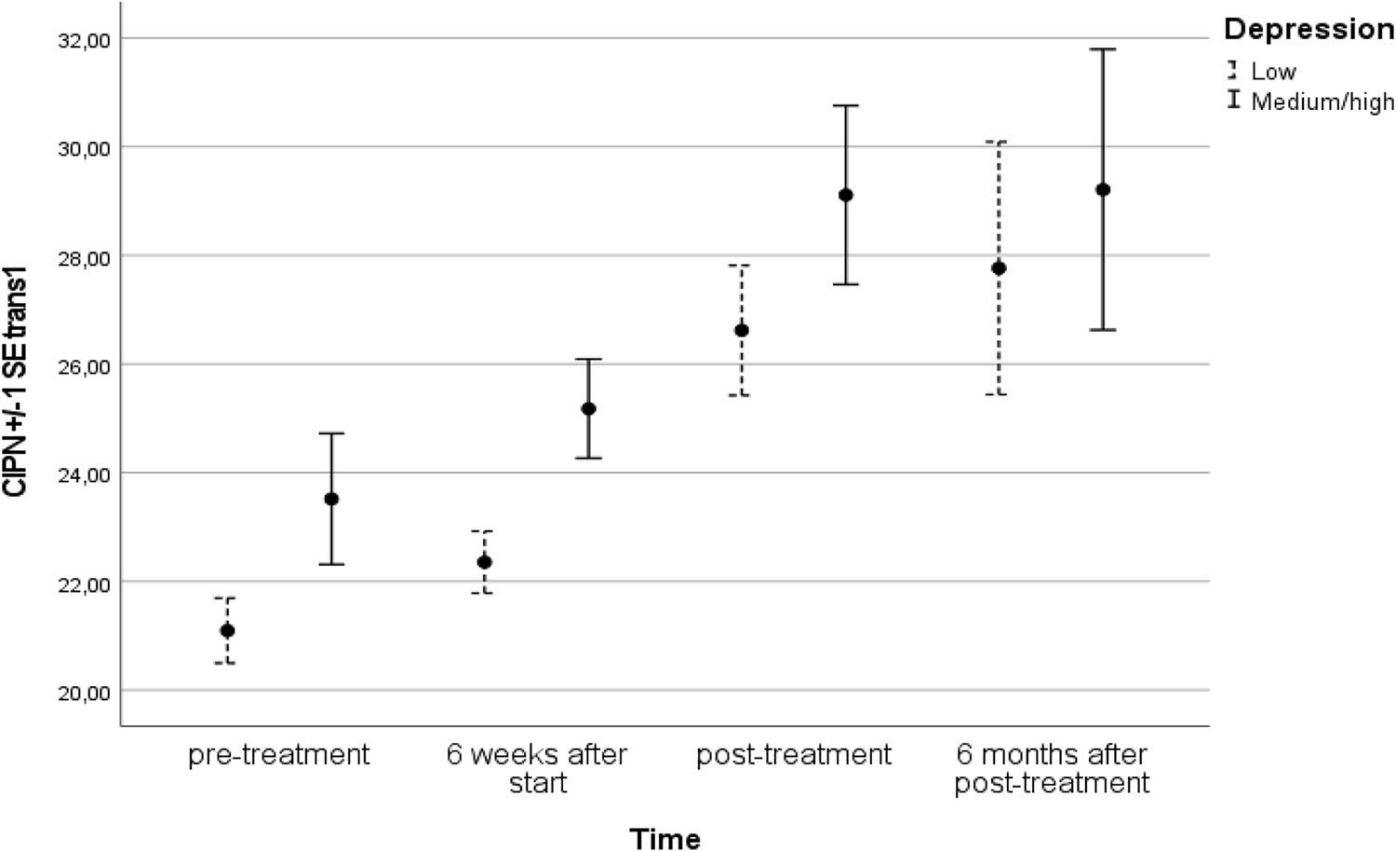
CIPN levels at 4 time points for group with low and medium/high depression at baseline

Similarly, we computed a conditional growth model with baseline level of depression. This model did fit the data better than the unconditional growth model (AIC = 1327.55) but showed that level of depression did not impact the development (*p* = .89) of CIPN (see also Figure 2).

## Discussion

This prospective study investigated the relationship between levels of anxiety and depression before the start of chemotherapy and the development and level of CIPN during and after treatment. Our results indicate that pre-treatment anxiety but not depressive symptoms may be a risk factor for developing CIPN in women with breast cancer during and after treatment with taxane-based (neo)adjuvant chemotherapy. These results are in line with Lee’s study that also found an association between pre-treatment anxiety and CIPN [15]. The present study adds to this finding by showing that more anxious patients experience more neuropathy before start and during chemotherapy and that, 6 months after ending chemotherapy, neuropathic symptoms increase even further. In contrast, less anxious patients start at a lower level of neuropathy and although showing an increase in neuropathic symptoms during chemotherapy this level is much lower than in the more anxious group and does not increase after ending chemotherapy.

Both psychological and biological effects of anxiety need to be considered when explaining the association between anxiety and the development of CIPN. Studies have shown that people with high levels of anxiety may be hypersusceptible to pain [19, 20], partly because they are more inclined to catastrophic thinking [21, 22]. Catastrophic thinking is characterized by attention to threat, overemphasis of the probability of a catastrophic outcome, and rumination about the worst possible consequences. It seems to play a unique role in the experience of higher levels of physical disability and negative physical sensations, like pain [23, 24], because it focuses the attention on the pain. This focus on pain may even be exacerbated when being informed by the medical specialist about the possible side effects of chemotherapy such as neuropathic symptoms. More anxious patients might be more suggestible to the negative expectations about getting pain (nocebo effect) which may function as a self-fulfilling prophesy [25, 26]. In addition to psychological mechanisms, biological factors may explain the relationship between anxiety and the development of more CIPN. More anxious patients may exhibit higher levels of proinflammatory cytokines interleukin-6 (IL-6) [27–29], which might interfere with recovery from the nerve injury in CIPN, resulting in more CIPN over time [30, 31]. Interestingly, in this study as well as in the study by Lee (2018), no relationship between depressive symptoms and the development of neuropathy was found. More research into the underlying mechanisms is needed to examine why pretreatment depression is not associated with CIPN severity and development while it has been related to more physical pain in other populations [32].

Neuropathy is a common adverse effect of taxane-based chemotherapy, but also of other anticancer drugs and may last for months and even years [33]. Given the increasing number of cancer survivors, there is an urgent need for prevention and treatment strategies for patients with CIPN. The burden experienced by neuropathy may impact optimal cancer treatment by limiting the dose of the anticancer drugs or discontinuing the treatment [2] and may worsen global quality of life and physical, role, cognitive, and social functioning compared to survivors without CIPN [34, 35]. To date, no treatment can be proposed as a gold standard to prevent or treat CIPN [7, 33, 36]. The results of this study might open the door for studies investigating interventions that may help prevent the development of excessive neuropathy by reducing pre-treatment anxiety. Till date, most psychological interventions focus on reducing or dealing with neuropathy after it developed [34, 37]. Whether psychological and/or pharmacological interventions with the aim of reducing anxiety before chemotherapy may have a positive impact on the development of CIPN should be investigated in future studies.

This study has several strengths such as the use of validated measures for CIPN, anxiety, and depression. For the degree of CIPN, we have used the EORTC-QLQ-CIPN20. This measure has a strong association with the often used NCI-CTCAE [38]. While both measures are validated, the QLQ-CIPN20 questionnaire provides more detailed information, distinguishes more subtle degrees of neuropathy, and is more responsive to change over time [4]. To detect subtle changes in level of neuropathy is especially important in the case of prevention. Moreover, CIPN was assessed four times in a 9-month period. Data was analyzed using mixed model analyses which are an extension of a linear regression model with the advantage that it focuses on individual patient’s patterns of scores through time rather than on mean values at each of the time points. A mixed model acknowledges that differences at later time points may be due to baseline effects and that the four separate scores over time by the same subject are correlated. A limitation of the present study is the relatively small sample size and short follow-up period. CIPN may develop months to years after ending chemotherapy [4, 15, 39]. Long-term research is required to investigate whether the effect of pre-treatment anxiety on the development of CIPN remains.

In conclusion, the level and persistence of CIPN after taxane-based chemotherapy seem to be associated with preexisting anxiety. Consequently, future studies may investigate the mechanisms by which anxiety influences CIPN and whether interventions targeting pre-treatment anxiety could lessen the development of CIPN.

## Data Availability

The datasets used and/or analyzed during the current study are available from the corresponding author on reasonable request.
